# Should we go nuts about nuts?

**DOI:** 10.1186/1741-7015-11-165

**Published:** 2013-07-16

**Authors:** Sabine Rohrmann, David Faeh

**Affiliations:** 1 Institute of Social and Preventive Medicine, University of Zurich, Hirschengraben 84, 8001, Zurich, Switzerland

**Keywords:** Health benefits, Mortality, Nuts, Prevention

## Abstract

Since the beginning of the 1990s, increasing evidence supports beneficial effects of nut consumption on health. A new analysis of the Spanish PREDIMED trial, published in *BMC Medicine*, has expanded our knowledge. The study showed that individuals eating nuts more than three times per week died less often from cardiovascular disease and cancer than non-consumers. The study also adds an important finding that previous epidemiological studies could not provide: a protective effect on premature mortality was only seen in the intervention group in which nut consumption increased during the 4.8 years of follow-up, not in the intervention group with additional olive oil consumption or in the control group. Nut consumption actually decreased during follow-up in the latter two groups. Questions remain to be answered on the quantity of nuts to be consumed for health benefits, on possible mechanisms of action, and on whether some types of nuts should be favored.

Please see related research: http://www.biomedcentral.com/1741-7015/11/164.

## Background

More than 20 years ago, the first studies were published on possible favorable effects of nut consumption on health in the general population [1,2]. Since then, the effects of nut consumption on health have been examined intensively. Nut consumption improves the body’s lipid profile and may influence inflammatory processes, oxidative stress, vascular reactivity and glycemic control [3]. The study by Guasch-Ferré *et al*. published in *BMC Medicine* which used information of the dietary intervention trial PREDIMED [4], provides additional convincing support for protective effects on cardiovascular disease and cancer mortality. Still, some questions remain open.

### Study results

Guasch-Ferré *et al*. [4] examined the association between consumption of almonds, peanuts, hazelnuts, pistachios, pine nuts and walnuts (as a separate group) and mortality in the PREDIMED nutrition intervention trials. The current analysis of the PREDIMED study included 7,216 individuals with increased cardiovascular risk [4]. Compared to never consumers of nuts at baseline, a decreased overall mortality was seen in those who consumed one to three servings (one serving = 28 g of nuts) per week (hazard ratio (HR) = 0.71, 95% confidence interval (CI) 0.54 to 0.83) and those who consumed three or more servings per week (HR = 0.61, 95% CI 0.54 to 0.93). An effect on overall mortality was observed for walnuts and ‘other types of nuts’. The effect was similar for cardiovascular mortality, whereas for cancer mortality an inverse association was observed only in those eating three or more servings and only for walnuts.

### Future directions

In 2003, a qualified health claim was approved by the U.S. Food and Drug Administration for nuts and cardiovascular disease, which states that ‘Scientific evidence suggests but does not prove that eating 1.5 ounces (42 g) per day of most nuts, as part of a diet low in saturated fat and cholesterol, may reduce the risk of heart disease.’ The European Union added in 2012 ‘Walnuts contribute to the improvement of the elasticity of the blood vessels.’ The claim may be used only for food that provides a daily intake of 30 g of walnuts. So, do we need any more evidence that nuts are good for health?

#### Isn't it all just due to the Mediterranean diet?

Although nuts are consumed by about 75% of the European population [5], the average daily consumption is small and follows a north to south gradient (0.4 g/day in the north and 1.7 g/day in the south [6]). Therefore, based on the PREDIMED study, one may simply conclude that protection rather comes from a general adherence to the Mediterranean diet than from isolated nut consumption. However, so far, most evidence suggesting that nut consumption may protect from cardiovascular and other chronic diseases comes from the US [3]. In the US, nut consumption is generally as low as in middle and northern European countries and there is only a weak adherence to the Mediterranean diet [7]. Nut consumption improves nutrient intake and diet quality [7]; thus, further evidence for an effect of nut consumption in addition to a generally healthy diet is warranted. Just recently [8], the PREDIMED trial showed that a Mediterranean diet supplemented with olive oil or nuts reduced the incidence of cardiovascular disease compared with a control diet in which the participants were told to reduce dietary fat intake. However, neither intervention significantly decreased all-cause or cardiovascular mortality, which were secondary outcomes of the PREDIMED trial. Thus, the current analysis of the PREDIMED data speaks in favor of an additional protective effect of nut consumption in the framework of a healthy diet [4,8].

#### Are walnuts particularly healthy?

The results of this study have shown an effect of any nut consumption on all outcomes [4]. However, although reduced all-cause and cardiovascular disease mortality were observed among consumers of walnuts and ‘other nuts’, cancer mortality was only inversely associated with the consumption of walnuts. Nuts are rich in polyunsaturated fatty acids, but also in minerals (calcium, magnesium, potassium) and vitamins. Additionally, they contain a number of phytochemicals such as phenolic acids, polyphenols and phytosterols [3,9]. Compared to other nuts, walnuts have a particularly high content of alpha-linolenic acid, and possibly also a higher bioavailability of the above-mentioned phytochemicals [10]: walnuts are usually consumed with the ‘skin’, which has the highest content of phytochemicals, whereas this skin is usually removed from other nuts before consumption. Thus, it has been speculated that walnuts might have stronger health effects than other types of nuts. This is however in contrast to other results, for example, from a meta-analysis showing that different types of nuts had similar effects on blood lipid levels [11]. So far, evidence is lacking for advice concerning a specific type of nut in addition to general nut consumption.

#### Health effects of nuts: do changes over time and overall duration of consumption matter?

Looking merely at the overall results of this study, there is a strong inverse association between baseline nut consumption and mortality. Looking more in depth shows that the dietary behavior during the follow-up period was important for the observed effects [4]. In fact, after disentangling the three arms, the association was only statistically significant in the arm with the nuts intervention (HR = 0.37, 95% CI 0.22 to 0.66), not in the olive oil intervention arm (HR = 0.63, 95% CI 0.36 to 1.10) or in the control arm (HR = 0.84, 95% CI 0.48 to 1.44). In this respect, it is interesting to note the changes in nut consumption in the three groups: participants in the Mediterranean diet arm with additional nuts increased their nut consumption by about 16 g/day. By contrast, consumption decreased by 0.8 g/day in the Mediterranean diet arm with olive oil and by 3.1 g/day in the control arm. To further clarify the question of changes in consumption over time in the context of other dietary properties, further research is required. It will, for example, be interesting to learn more about the impact of timing and duration of nut consumption on cancer incidence given that some types of cancer develop with a latency of decades.

#### What amount has to be consumed to provide health benefits?

Guasch-Ferré *et al*. observed an effect in a Mediterranean country [4], where nut consumption is usually higher than in other Western countries [6]. An overall protective effect was observed for those consuming at least one to three servings per week, which translates to approximately 4 to 12 g/day; for cancer, the effect was observed only among those who consumed three or more servings per week. This is in line with results from other studies [12,13]. In an Australian study [14], a statistically significant reduction in death due to inflammatory causes was observed for as little as 1.4 g/day compared with < 0.9 g/day. However, this leads to one of the crucial questions in nutrition epidemiology: how validly can nut consumption be assessed and monitored in large epidemiological studies? The question of how many nuts do we need to eat for how long to prevent x, y and z may best be addressed in intervention studies, in which consumption can be better controlled and assessed than in observational studies.

## Conclusion

The analysis by Guasch-Ferré and colleagues contributes further evidence that nut consumption is good for health and may prevent premature deaths. Questions about specific constituents, amount, duration and type of nuts to be consumed remain to be elucidated. Meanwhile, we might need to focus on the question of how to better promote nut consumption in the population and sustainably integrate it into the daily diet. Currently, several dietary guidelines recommend replacing one of five servings of fruits and vegetables a day by a serving of nuts. This appears to be a simple and practical recommendation to start with.

## Competing interests

The authors declare that they have no competing interests.

## Authors' contributions

SR drafted the manuscript and DF critically revised the content. Both authors read and approved the final manuscript.

## Authors' information

SR is a nutritional epidemiologist whose research focuses on diet and lifestyle as risk factors of cancer. She is Assistant Professor of Chronic Disease Epidemiology and head of the Division of Cancer Epidemiology and Prevention at the Institute of Social and Preventive Medicine at Zurich University. DF is physician and epidemiologist with a focus on cardiovascular disease. He is a scientist and lecturer at the Institute of Social and Preventive Medicine at Zurich University.

## Pre-publication history

The pre-publication history for this paper can be accessed here:

http://www.biomedcentral.com/1741-7015/11/165/prepub
